# Molecular evidence of hookworms in public environment of Bangladesh

**DOI:** 10.1038/s41598-022-26813-8

**Published:** 2023-01-04

**Authors:** Tilak Chandra Nath, Keeseon S. Eom, Seongjun Choe, Hansol Park, Dongmin Lee

**Affiliations:** 1 International Parasite Resource Bank, Chungbuk National University, Cheongju, South Korea; 2 Department of Parasitology, Sylhet Agricultural University, Sylhet, Bangladesh; 3 Department of Parasitology and Parasite Research Center, School of Medicine, Chungbuk National University, Cheongju, South Korea

**Keywords:** Parasite biology, Parasite development, Risk factors

## Abstract

Accurate diagnosis by precise identification of causative agents is essential for the effectiveness of any control interventions. Despite high zoonotic potential, available literature on hookworms in Bangladesh is still scarce and nonspecific. The objective of this study was to determine the occurrence of hookworms in public locations across northeastern Bangladesh (Sylhet metropolitan area) using integrated parasitological and molecular assays. A total of 130 samples (80 soil and 50 environmental canine feces) were collected and examined using modified flotation technique and formalin-ether sedimentation methods. Modified plate culture was used to isolate larvae. The identification was made based on morphometric features and confirmed by amplifying the ITS region of the nuclear rDNA. Overall, 66.2% (86/130) of examined samples were positive for hookworms infection. Characteristic eggs (61–68 × 29–37 μm) and/or larvae of hookworms were observed in 73.8% (59/80) soils and 54.0% (27/50) environmental fecal samples. Rhabditiform larvae (0.48–0.54 × 0.04–0.07 mm) were observed in cultured samples. Genetic analysis of rDNA sequences revealed the presence of *Ancylostoma caninum* and *Ancylostoma ceylanicum.* In this study, hookworms' contamination of the public environment was substantial. To the best of our knowledge, this is the first molecular proof of *A. caninum* and *A. ceylanicum* observed in urban public environment in Bangladesh.

## Introduction

Contaminated public environments with zoonotic helminths represent a risk for human health, however, often overlooked for funding and research. Hookworms, belong to the family Ancylostomatidae, is one of the most common soil-transmitted helminths (STHs), infect mammals including humans^1^. Hookworms infection has been documented globally, especially in temperate and tropical regions with low-socioeconomic status. Hookworms infect approximately one-tenth of the population in the Indian subcontinent, mostly school-aged children and pregnant women^2^. It showed that several areas, especially less developed urban areas had a high prevalence of hookworm infections. According to a recent small-scale assessment conducted in three regions of Bangladesh, dogs were observed as a reservoir for hookworm infections, with an overall prevalence of 79.1% found in feces^3^. Climatic conditions like humidity, temperature, and rainfall greatly influence the epidemiology of hookworms^2^. Currently, more than 100 species belonging to 18 genera of hookworms have been reported, parasitizing the intestinal tract of respective hosts. Hookworms belong to the Ancylostomatidae family can infect both humans and animals. *Ancylostoma duodenale* and *Necator americanus* are the most common hookworm species found in humans. Besides these, zoonotic infection with several other species like *A. caninum, A. braziliense, and A. ceylanicum* has been reported in many regions of Asia and Southeast Asia^4^. Although most infections with these nematodes remain asymptomatic by the host, high worm loads, especially in children and pregnant women, may cause severe complications such as anemia, dyspepsia, abdominal pain, diarrhea, dietary deficiencies, and delayed physical and cognitive development^4,5^. Children, pregnant women, and immunocompromised peoples are more susceptible to hookworms infection^3^. Human acquires infection via infective larvae that can penetrate intact skin.

*A. caninum* and *A. ceylanicum* are blood-feeding canine hookworms, infecting the host through contact with the 3^rd^ stage larvae via cutaneous route or ingestion of foods contaminated with the eggs/larvae^4,6^. Both species utilize dogs and cats as their natural definitive hosts. Besides their veterinary importance, both species are known to cause patent infection in humans^7^. Human exposure is more common in areas where animal feces may not be properly disposed. Certain behaviors like walking barefooted, exposure to soil, and animal cohabitation might increase the risk of transmission. Although hookworm infections continue to be a major health problem, the role of the environment as a reservoir for hookworm infections in humans and animals remains poorly explored and available information on hookworms is scarce. The main reason for this paucity of demographic information is that hookworms infection is often diagnosed by demonstrating eggs. Since all hookworm species have eggs that are morphologically identical, conventional diagnostic methods cannot distinguish between hookworm species. Detecting specific hookworm is important for several reasons. Firstly, among STHs, hookworms are considered successful parasites because they can infect their hosts through multiple routes. Second, despite the fact that the majority of commonly used anthelmintics are effective against hookworms, anthelmintic resistance to hookworms has been observed on global scale in recent years^6^. Therefore, this study combined traditional parasitological techniques with molecular methods to unfold the presence of zoonotic hookworm species in public environments.

## Methods

Samples were collected from different local spots within Sylhet metropolitan area (Lakkatura, Tilagor, Bondor, and Varthakhola), Bangladesh which have been recognized as soil-transmitted helminths endemic areas^8^. Using a cross-sectional study design and purposive sampling approach, 80 soils and 50 canine fecal samples that had been defecated in public areas were collected between January and June 2021. For each case, about 50 g of soil samples at a depth of 2–3 cm of topsoil were collected in zippered polyethylene bag, and about 5 g of feces samples were collected in screw-capped container. Parasitological investigations were done at the Department of Parasitology, Sylhet Agricultural University, Bangladesh, while molecular analysis was carried out at the Department of Parasitology, Chungbuk National University, Republic of Korea.

Collected samples were initially examined on the same day of collection. Parasitological assessment of soil samples was processed using the modified flotation method (Sheather's sugar flotation), whereas the fecal samples were treated using the formalin-ether sedimentation technique. The specific gravity of the flotation solution was adjusted to 1.27 and a small quantity of 10% formalin was added to the prepared flotation solution to facilitate transitory preservation^8^. Observed larvae from soil and fecal samples were collected using pipette under stereomicroscope (Olympus ACH-1X, Japan) and preserved in fixatives (10% formalin and 95% ethanol). DNA extraction from soil samples was done following a previously described protocol with slight modification^7^. The flotation procedure of soil samples was repeated 3 times and 3 ml of top layered supernatant was collected in a separate test tube using pipette. The collected supernatant-containing tube was mixed with distilled water and then centrifuged at 1500 rpm for 5 min. The sediment was then processed using the Qiagen Blood and Tissue kit (Qiagen, Hilden, Germany) according to manufacturer’s instructions. For DNA exaction from feces, positive fecal samples were cultured for 6 to 9 days using the plate culture method with modification^9^. The cultured samples were moistened with physiological saline and checked daily for 9 days with a stereomicroscope. Total genomic DNA from isolated larvae was extracted by using a commercial kit (DNeasy Blood and Tissue Kit, Qiagen, Germany). When larvae were observed, it was collected and preserved. For morphological observation, the worms were placed in lactophenol, and measurements were made with a light microscope (Olympus BX-53, Japan). The rDNA spanning ITS was amplified using NC5: 5'-gtaggtgaacctgcggaaggatcatt-3', NC2: 5'-ttagtttcttttcctccgct-3' and NC1:5′-acgtctggttcag ggttgtt-3ʹ, NC2: 5ʹ-ttagtttcttttcctccgct-3ʹ primer sets^10^. The PCR reactions were performed in a Kyratec PCR Thermal Cycler (Queensland, Australia). The PCR reactions applied the following cycling profile: initial denaturation of 95 °C for 15 min, followed by 30 cycles of denaturation, annealing, and extension at 95 °C for 45 s, 55 °C for 45 s, and 72 °C for 45 s, respectively. The final extension was carried out at 72 °C for 10 min. Then, the PCR products were checked using 1.5% agarose gel and visualized under UV light. DNA sequencing was performed by Cosmogenetech, South Korea. Of positive samples, 3 soil samples and 3 fecal samples were randomly selected for DNA sequencing and subsequent phylogenetic analysis. The obtained sequences were assembled with Geneious program 9.0 (Biometer, New Zealand). Sequences were aligned using ClustalW multiple alignments implanted MEGA7^11^. Sequencing was carried out by BLAST algorithms and databases from the National Center for Biotechnology Information database. Phylogenetic trees were constructed using maximum likelihood (ML) algorithms with bootstrap values calculated using 1000 replicates.

All methods were performed in accordance with the relevant guidelines and regulations by Parasite Research Center and Parasite Resource Bank, Chungbuk National University, South Korea, and the Department of Parasitology, Sylhet Agricultural University, Bangladesh. No experimental animals and human participants were involved in this study.

### Ethical approval and consent to participate

This study protocol was reviewed and approved by the Parasite Research Center and Parasite Resource Bank, Chungbuk National University, South Korea (2020EC-59), and the Department of Parasitology, Sylhet Agricultural University, Bangladesh. Informed consent for participation was not applicable in this study.

## Results and discussion

**Figure 1:**
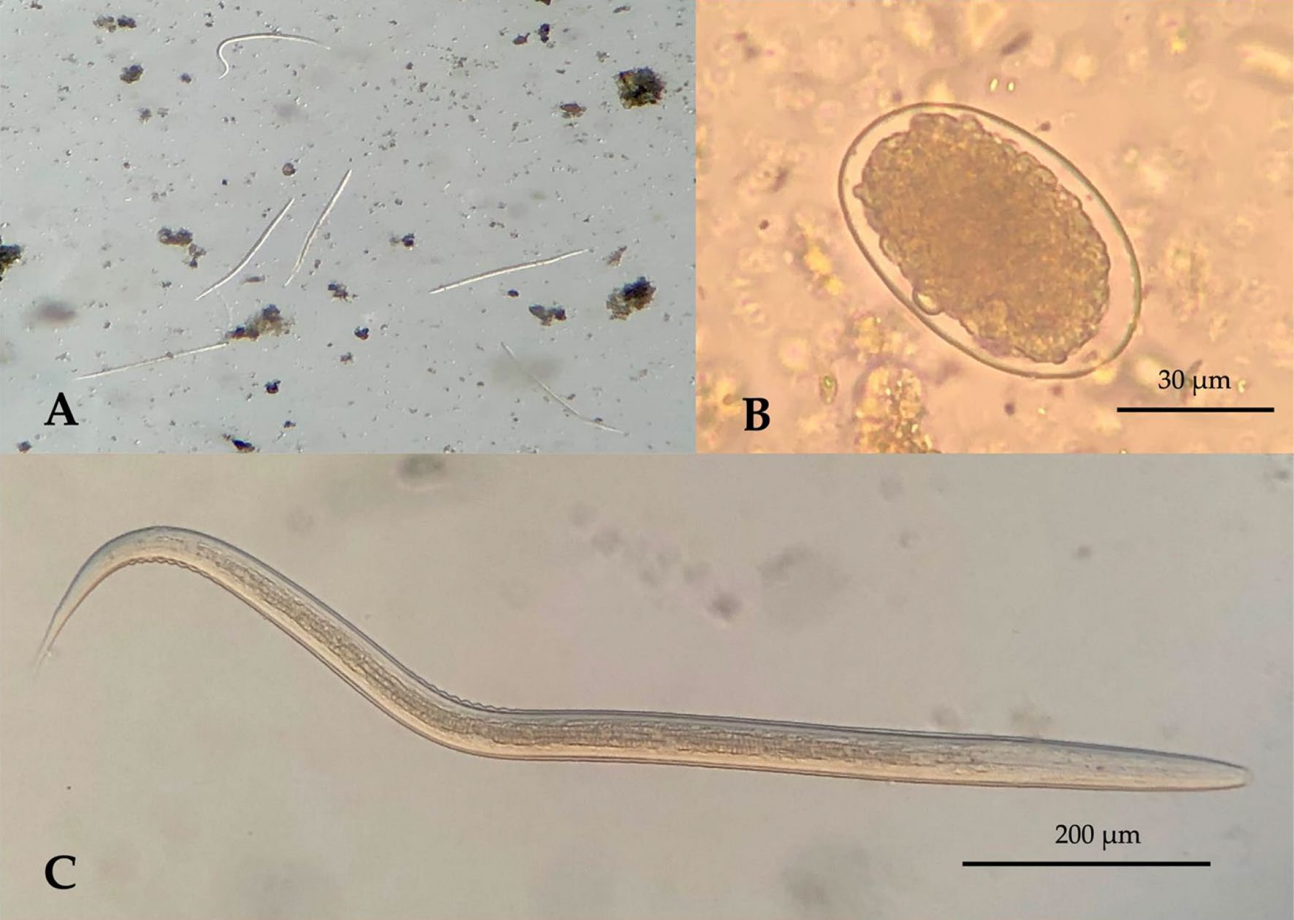
Photomicrographs of hookworms’ larvae and egg, (**A**) cultured larvae from stool sample, (**B**) egg, (**C**) cultured rhabditiform larvae.

Overall, 59 of 80 soil samples (73.8%) and 27 of 50 environmental fecal samples (54.0%) were positive by microscopic examination (Table 1). Eggs appeared oval, were covered with a thin envelope, and were 61–68 µm in length and 29–37 µm in width (Fig. 1B). Of the positive stool samples, depending on the incubation time, larvae were observed (Fig. 1A). They are approximately 0.48–0.54 mm in length and 40–70 μm in width (Fig. 1C). The larvae are rhabditiform, the tail is short without filament and tapers sharply that it resembles a pencil point. While there is some variation, the measurements of most hookworm species are very similar and cannot be differentiated.

**Table 1:** Prevalence of hookworms in public environment of Sylhet, Bangladesh.

	Samples examined	Positive	Negative	p-value*
	N	n (%)	n (%)	
Soil	80	59 (73.8%)	21 (26.2%)	0.002
Canine feces	50	27 (54.0%)	23 (46.0%)	
Total	130	86 (66.2%)	44 (33.8%)	

*p-value from Chi-square test.

**Figure 2:**
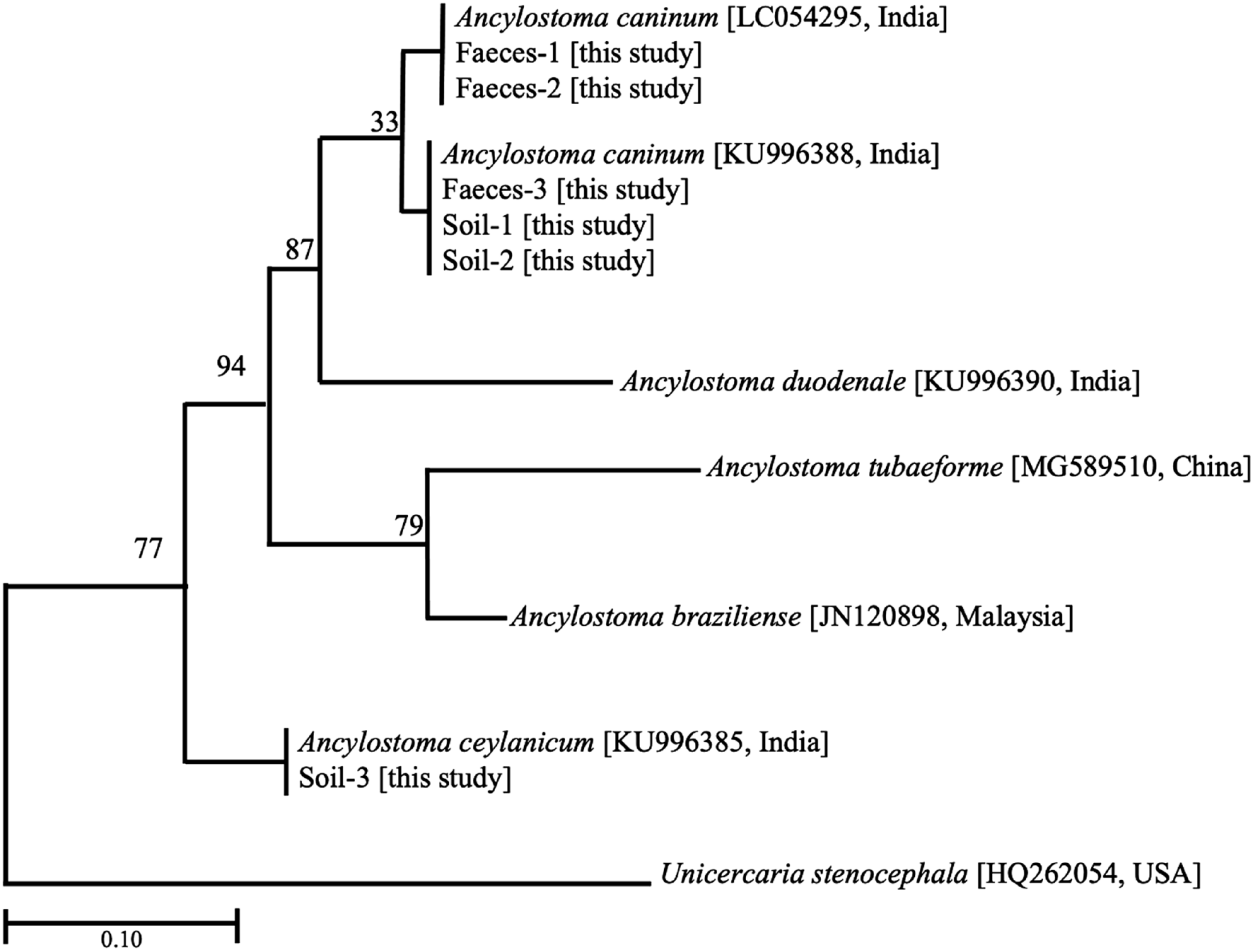
Phylogenetic tree of hookworm isolates reconstructed by maximum-likelihood method based on the ITS sequences to draw inferences on the relationship between different species. The reference sequences are available in the GenBank by their accession numbers. Numbers at the branch nodes indicate the degree of bootstrap support for 1000 replicates.

Due to similar size and morphology, hookworms’ eggs and larvae are difficult to differentiate by microscopy and the use of molecular technique like PCR has proven effective to differentiate species. In the present study, fragments of larvae (soil-derived isolates and feces-derived isolates) were successfully amplified (706–719 bp) and assembled. Sequencing analysis revealed 99.4–100% similarity to the reference sequences of *Ancylostoma caninum* (GenBank number LC054295, KU996388) and 99.1% similarity to the reference sequences of *Ancylostoma ceylanicum* (GenBank number KU996385). Phylogenic tree constructed using ML methods also showed the isolated hookworms in the present study genetically clustered into two distinct groups with *A. caninum* and *A. ceylanicum* (Fig. 2).

To determine the public health risk posed by zoonotic parasites, environmental contamination with helminth infective stages must be investigated. In our study, 66.2% (86/130) of examined samples were found positive and two known hookworm species which were zoonotic hookworms (*A. caninum* and *A. ceylanicum*) species were found. Contaminated soils with canine feces are significant sources of diseases for humans, animals, and the community at large^12^. Dogs and cats are the most prevalent domestic animals in Bangladesh, and they frequently roam freely and defecate in public places^3^. Additionally, the moist soils in these regions enhance the development and survival of infectious stage larvae, aggravating hookworms spread. Human infection with zoonotic hookworms, especially by *A. ceylanicum* was reported in several countries like Thailand, Taiwan, Philippines, Laos, and the neighboring country India^7^. Canine hookworm infection is endemic in South Asian countries with a prevalence ranging from 70 to 100%, and zoonotic transmission of the disease is a potential source of serious public health issues^13^. Infected eggs or larvae in the environment play a critical role in spreading intestinal nematodes in both humans and animals^14^. In the present study, a large proportion of examined samples were found to contain *A. caninum* DNA. Through microscopic analysis of dog feces from several districts of Bangladesh, Singh et al.^12^ and Nath et al.^15^ found that hookworms prevalence was 79.1% and 78.9%, respectively. Contaminated soil was reported as the main route by which hookworms infect humans, however, negligence in performing diagnostics is common in endemic disadvantaged communities^16^. In livestock, the unauthorized use and complete reliance on anthelmintic drugs for control of parasitic infections have led to high levels of anthelmintic resistance. In addition to animal health issues, multiple drug resistance in hookworms might pose major public health threats since some hookworms are zoonotic^14,17^. In these circumstances, the link between physicians, veterinarians, and the entire community should be strengthened to minimize the likelihood of a public hazard.

The information presented here provides interesting insights into environmental contamination of zoonotic hookworms in Sylhet, utilizing molecular diagnostics for the first time in Bangladesh. Due to the widespread presence of *A. ceylanicum* and *A. caninum* in public areas, people are at a greater chance of acquiring infections. One Health approach, which includes mass deworming programs for canines, regular inspections for the presence of helminths eggs in dog premises soil, and raising public awareness through health education, should be emphasized. This study also highlights the need for additional investigation of hookworm genotyping in different hosts and diverse environmental settings.

## Data Availability

The datasets and resources used in this current study are available from the first author upon reasonable request.

## Abbreviations

STHs: Soil-transmitted helminths
rDNA: Ribosomal deoxyribonucleic acid
ITS: Internal transcribed spacer
ML: Maximum likelihood
BLAST: Basic local alignment search tool
PCR: Polymerase chain reaction
MEGA: Molecular evolutionary genetics analysis
