# Author Correction: Molecular evidence of hookworms in public environment of Bangladesh

**DOI:** 10.1038/s41598-023-31506-x

**Published:** 2023-03-21

**Authors:** Tilak Chandra Nath, Keeseon S. Eom, Seongjun Choe, Hansol Park, Dongmin Lee

**Affiliations:** 1 International Parasite Resource Bank, Chungbuk National University, Cheongju, South Korea; 2 Department of Parasitology, Sylhet Agricultural University, Sylhet, Bangladesh; 3 Department of Parasitology and Parasite Research Center, School of Medicine, Chungbuk National University, Cheongju, South Korea

Correction to: *Scientific Reports* 10.1038/s41598-022-26813-8, published online 04 January 2023

The original version of this Article contained an error in the Funding section,

“This work was supported by Basic Science Research Program through the Chungbuk Techno-Park funded by Chungcheongbuk-do, Korea. The funders had no role in the study design, analysis, decision to publish, or preparation of the manuscript.”

now reads:

“This research was supported by ‘Regional Innovation Strategy (RIS)’ through the National Research Foundation of Korea (NRF) funded by the Ministry of Education (MOE) (2021RIS-001).”

The original Article has been corrected.

